# Comprehensiveness of cardiac rehabilitation program in Korea: a nation-wide survey result

**DOI:** 10.1186/s12872-023-03204-z

**Published:** 2023-04-06

**Authors:** Jidong Sung, Chul Kim, Jae-Young Han, Sungju Jee, Jang Woo Lee, Jong Hwa Lee, Won-Seok Kim, Heui Je Bang, Sora Baek, Kyung-Lim Joa, Ae Ryoung Kim, So Young Lee, Jihee Kim, Chung Reen Kim, Oh Pum Kwon

**Affiliations:** 1 Division of Cardiology, Department of Medicine, Sungkyunkwan University School of Medicine, 115 Irwon-ro, Gangnam-gu, Seoul, 06355 Korea; 2 Department of Rehabilitation Medicine, Inje University Sanggye Paik Hospital, Seoul, 01757 Korea; 3 Department of Physical Medicine and Rehabilitation, Chonnam National University Medical School and Hospital, Gwangju, 61469 Korea; 4 Department of Rehabilitation Medicine, Chungnam National University College of Medicine, Daejeon, 35015 Korea; 5 Department of Physical Medicine and Rehabilitation, National Health Insurance Service Ilsan Hospital, Goyang, 10444 Korea; 6 Department of Physical Medicine and Rehabilitation, Dong A University College of Medicine, Busan, 49201 Korea; 7 Department of Rehabilitation Medicine, Seoul National University Bundang Hospital, Seongnam, 13620 Korea; 8 Department of Rehabilitation Medicine, Chungbuk National University Hospital, Cheongju, 28644 Korea; 9 Department of Rehabilitation Medicine, Kangwon National University School of Medicine, Chuncheon, 24289 Korea; 10 Department of Rehabilitation Medicine, Inha University Hospital, Incheon, 22332 Korea; 11 Department of Rehabilitation Medicine, School of Medicine, Kyungpook National University, Daegu, 41944 Korea; 12 Department of Rehabilitation Medicine, Jeju National University School of Medicine, Jeju, 63241 Korea; 13 Department of Rehabilitation Medicine, Wonkwang University School of Medicine, Iksan, 54538 Korea; 14 Department of Rehabilitation Medicine, Ulsan University College of Medicine, Ulsan, 44033 Korea

**Keywords:** Cardiac rehabilitation, Comprehensive health care, Health education

## Abstract

**Background:**

Cardiac rehabilitation (CR) is an essential component in secondary prevention of cardiovascular diseases. Current guidelines recommend that the program should be comprehensive including multidisciplinary behavioral intervention, not only exercise training. While the utilization of CR is gradually increasing, the comprehensiveness of the program has not been systemically evaluated in Korea.

**Methods:**

During the year 2020, nation-wide survey was done to evaluate the current status of CR in Korea. Survey was done by web-based structured questionnaire. Survey was requested to 164 hospitals performing percutaneous coronary intervention.

**Results:**

Among 164 hospitals, 47 (28.7%) hospitals had CR programs. In hospitals with CR, multidisciplinary intervention other than exercise-based program was provided only partially: nutritional counseling (63%), vocational counseling for return to work (39%), stress management (31%), psychological evaluation (18%). Personnel for CR was commonly not dedicated to the program or even absent: (percentage of dedicated, concurrent with other work, absent) physical therapist (59, 41, 0%), nurse (31, 69, 0%), dietician (6, 65, 29%), clinical psychologist (0, 37, 63%).

**Conclusion:**

Comprehensiveness of CR in Korea is suboptimal and human resource for it is poorly disposed. More awareness of current status by both clinicians and health policy makers is needed and insurance reimbursement for educational program should be improved.

## Introduction

Cardiac rehabilitation (CR) is being recommended as an essential component of secondary prevention of cardiac diseases such as coronary artery disease and heart failure by guidelines [1–3]. Though evidences for benefits of CR has been mainly established from the results of the studies of exercise-based CR program [4] and exercise training is effective and essential core component of CR, [5] current guidelines and experts recommend comprehensive program integrating various lifestyle interventions such as delivery of dietary advice, smoking cessation program and cognitive behavioral therapy to improve psychosocial health, in addition to exercise training [1, 6]. A meta-analysis including recent trials of CR reported that comprehensive programs managing six or more risk factors reduced all-cause mortality while those managing less did not [7].

Experts have pointed out various barriers in implementing comprehensive CR and among which is a personnel factor [8]. Working in multidisciplinary teams were suggested as a core competency [9]. Recently published Korean guideline for cardiac rehabilitation clearly stated that CR programs is not just exercise training and should include patient education to ensure the comprehensiveness of the program [10]. However, comprehensiveness of Korean CR programs has not been adequately studied and reported.

The purpose of this study is to investigate and describe the comprehensiveness of CR program and its human resource disposition using Korean nation-wide survey data.

## Methods

### Study subjects and performance of the survey

This is a descriptive study of current status based on the nation-wide survey data. To examine CR’s current status in the South Korea, the CR-General Questionnaire (CRGQ) was developed after analyzing the national and international CR clinical practice guidelines [1–3, 10] and the Cardiac Rehabilitation-In Depth Questionnaire (CR-IDQ) was developed with reference to the CR evaluation tools of York University [11, 12] and International Council of Cardiovascular Prevention and Rehabilitation [13]. During the year 2020, nation-wide survey was done to evaluate the current status of CR in Korea. Survey was done by web-based structured questionnaire. Direct field survey was impossible due to the COVID-19 pandemic. Survey questions regarding the details of the CR programs were directed to the personnel responsible for administration of CR programs. Detail of the survey process has been described elsewhere [14]. Survey results from 164 hospitals performing percutaneous coronary intervention (PCI) were analyzed.

### Components of CR program

Survey questions regarding the components of CR programs asked about whether following components were being provided or not: cardiovascular(CV) risk factor evaluation, cardiopulmonary function test (or other tests for exercise capacity), evaluation for comorbidity, individualized exercise prescription, supervised exercise session, education for self-monitoring of heart rate, education for CV risk factor management, medication adherence, nutritional counseling, psychological assessment, vocational counseling, stress management, reassessment at the end of the hospital-based program and follow-up after the program. Provided CR components were compared according to the regions: Seoul and Kyunggi vs. other regions. In Korea, medical resources are more concentrated in Seoul and Kyunggi region, the capital area, than the other regions. The numbers of hospitals with CR program are similar between Seoul and Kyunggi versus other regions. The comparison as such may reveal the characteristics of the capital area.

For hospitals without CR program, shortened questionnaire was done including questions whether following components were provided or not: cardiovascular (CV) risk factor evaluation, risk assessment for exercise, individualized exercise prescription, education for CV risk factor management, medication adherence, nutritional counseling.

### Disposition of human resource

Questions regarding the disposition of human resource were asked to the hospitals with CR program, asking whether personnel of the following discipline were available or not: medical director, physical therapist, registered nurse, clinical psychologist, dietician, exercise specialist. And when the personnel were available, it was checked whether the job of the position was dedicated to cardiac rehabilitation program or concurrent with other work.

### Statistical analysis

The proportions were reported in percent. The chi-square test was used to compare the proportions between regions. All statistical analyses were performed using the Statistical Package for the Social Sciences (SPSS) (version 19.0; SPSS Inc., Chicago, IL, USA). The level of significance was set at *p* < 0.05.

### Ethics statement

This study was approved by the Institutional Review Board of the Samsung Medical Center, Seoul, Korea (IRB No 2020-04-017) and other participating centers. The need to obtain informed consent was waived due to the non-clinical and minimal-risk nature of the study. No personal information (patient’s name, address, ID, phone number, hospital ID) were collected and thus the participants’ anonymity was preserved.

## Results

### Overall status and distribution of CR programs

Among the 164 PCI-performing hospitals nation-wide, 47 (28.7%) hospitals had established CR programs, which was reported in previously published article [14] but reproduced in brief in Table 1 for convenience. Both PCI-performing hospitals and centers with CR programs were concentrated in Seoul and Gyeonggi regions.

**Table 1:** Number and Distribution of Hospitals Performing PCI and CR by Region in Korea

Region	PCI Hospital, n	PCI Hospital nwith CR, n (%)	Annul No. of IHD *	Annual No. of AMI *
Seoul	31	13 (42)	254,753	25,715
Gyeonggi	33	9 (27.3)	184,453	23,834
Incheon	9	3 (33.3)	48,834	5926
Gangwon	5	2 (40)	31,748	4913
Daejeon-Chungnam	12	1 (8.3)	62,004	8204
Chungbuk	6	1 (16.7)	27,432	3254
Gwangju-Jeonnam	11	2 (18.2)	73,777	9373
Jeonbuk	5	1 (20)	32,372	5091
Busan	17	4 (23.5)	84,344	8674
Gyeongnam	11	3 (27.3)	51,940	7714
Ulsan	5	1 (20)	17,988	2004
Daegu-Gyeongbuk	15	6 (40)	93,054	15,402
Jeju	4	1 (25)	10,563	915
Total	164	47	943,006	118,872

CR: cardiac rehabilitation, PCI: percutaneous coronary intervention, IHD: ischemic heart disease, AMI: acute

myocardial infarction

* Data from Healthcare Big Data Hub, open data on high-interest diseases, 2019 (opendata.hira.or.kr)

Among 164 PCI-performing hospitals, 147 hospitals responded to the survey (Response rate 89.6%) and data from the 147 hospitals (47 with CR and 100 without CR) were used for analysis of this study. Among 47 hospitals with CR program, 91.8% were doing coronary artery bypass graft surgery, 41.2% were doing left ventricular assistance device and/or heart transplantation, in addition to PCI (100%). Among 51 medical directors of the CR program 82.4% were in the department of rehabilitation medicine, 11.8% in cardiology. The number of patients participating inpatient CR program was over 300 persons/year in 21.6%, 100–299/year in 35.2%, less than 100/year in 35.3%. However, the number of outpatient CR program is much lower: < 50/year in 45.1%, 50–99/year in 15.7%, ≥ 100/year in 31.4%.

While all the hospital with CR program had facility and equipment such as space for exercise therapy and treadmills, designated space for patient education was available only in 43%, 23% of the hospitals had the educational space used concurrently for other purpose, and absent in 12%.

### Components of CR program provided

In-depth questionnaire administered in 51 medical directors of 47 hospitals with CR program. Results are shown according to the hospitals. Table 2 show CR components provided. While all or majority of centers were providing components typically related to exercise-based CR program such as exercise prescription and supervised exercise training session and basic education components such as education for CR risk factor management and medication adherence, several components related to ‘comprehensive’ CR program such as nutritional counseling, psychological assessment, vocational counseling and stress management were less available. These were compared by regions, Seoul and Gyeonggi regions vs. other regions in Table 3, which showed no significant difference by regions.

**Table 2:** CR components provided in hospitals with CR program (N = 47)

CR components	Provided, n (%)	Not provided, n (%)
CV risk factor evaluation	46 (98)	1 (2)
Cardiopulmonary function test	46 (98)	1 (2)
Other tests for exercise capacity	39 (83)	8 (17)
Evaluation for comorbidity	34 (72)	13 (28)
Individualized exercise prescription,	47 (100)	0 (0)
Supervised exercise session	45 (96)	2 (4)
Education for self-monitoring of heart rate	44 (94)	3 (6)
Education for CV risk factor management	45 (96)	2 (4)
Medication adherence	43 (91)	4 (9)
**Nutritional counseling**	**29 (62)**	**18 (38)**
**Psychological assessment**	**8 (17)**	**39 (83)**
**Vocational counseling**	**17 (36)**	**30 (64)**
**Stress management**	**13 (28)**	**34 (72)**
Reassessment at the end of the hospital-based program	44 (94)	3 (6)
Follow-up after the program	41 (87)	6 (13)

CR: cardiac rehabilitation, CV: cardiovascular

**Table 3:** CR components provided according to regions (N = 47)

CR components	Seoul & Gyeonggi (n(%), N = 24)	Other regions (n(%), N = 23)	p*
CV risk factor evaluation	24 (100)	22 (96)	0.98
Cardiopulmonary function test	23 (96)	23 (100)	1.00
Other tests for exercise capacity	20 (83)	19 (83)	1.00
Evaluation for comorbidity	17 (71)	17 (74)	1.00
Individualized exercise prescription,	24 (100)	23 (100)	0.88
Supervised exercise session	23 (96)	22 (96)	1.00
Education for self-monitoring of heart rate	23 (96)	21 (91)	0.97
Education for CV risk factor management	23 (96)	22 (96)	1.00
Medication adherence	23 (96)	20 (87)	0.57
Nutritional counseling	17 (71)	12 (52)	0.31
Psychological assessment	4 (17)	4 (17)	1.00
Vocational counseling	10 (42)	7 (30)	0.62
Stress management	9 (38)	4 (17)	0.22
Reassessment at the end of the hospital-based program	22 (92)	22 (96)	1.00
Follow-up after the program	20 (83)	21 (91)	0.70

CR: cardiac rehabilitation, CV: cardiovascular

* between Seoul/Gyeonggi and other regions, by χ^2^ test

Table 4 shows educational components provided by hospitals *without* CR program. Despite not having CR program, these hospitals were providing some educational components such as CV risk factor evaluation, education for risk factor management and medication adherence in slightly lower frequency compared to the hospitals with CR program. Education for exercise was provided in less than one-third and offering of nutritional counseling was in similar proportion, compared to the hospitals with CR program.

**Table 4:** Educational components provided in hospitals without cardiac rehabilitation program (lnN = 100)

Educational components	Provided, n (%)	Not provided, n (%)
CV risk factor evaluation	77 (77)	21 (21)
Risk estimation for exercise	26 (26)	72 (72)
Individualized exercise prescription,	18 (18)	80 (80)
Education for CV risk factor management	79 (79)	19 (19)
Medication adherence	92 (92)	6 (6)
Nutritional counseling	60 (60)	38 (38)

CV: cardiovascular

### Disposition of human resource

While medical director (either rehabilitative medicine physician, cardiologist or cardiac surgeon), physical therapist and CR nurse (‘essential’ personnel designated by insurance reimbursement criteria) were available in 100% of cases, clinical psychologist were not available in 60% and dietician in 26%. It is rare that clinical psychologist and dietician were appointed to dedicated working positions for CR. Even the personnel considered essential were not entirely in dedicated position but doing other work concurrently in considerable proportion. (Table 5)

**Table 5:** Disposition of cardiac rehabilitation personnel (N = 47)

	Dedicated for CR	Concurrent position	Not available
Medical director	14 (30)	33 (70)	0 (0)
Physical therapist	27 (57)	20 (43)	0 (0)
CR nurse	19 (40)	28 (60)	0 (0)
Clinical psychologist	0 (0)	19 (40)	28 (60)
Dietician	2 (4)	33 (70)	12 (26)
Exercise specialist	1 (2)	6 (13)	40 (85)

CR: cardiac rehabilitation

## Discussion

This is the first study to evaluate the comprehensiveness of Korean CR program by nation-wide data. Study finding shows that current CR programs in Korea are mostly exercise-based program and its comprehensiveness is rather limited. Components such as nutritional counseling, psychological assessment, vocational counseling, and stress management were not adequately addressed and personnel for these components were not sufficient. While there already have been several studies to grasp the status of Korean CR program, most of them had some limitation in its scope [15–18]. This nation-wide survey project is more thorough and inclusive of various aspects of CR and with larger sample size and part of the survey results has been recently published, in which the detail of the research methods was described [14].

Implementing comprehensive CR program is a challenging task world-wide, not only in Korea. In a survey study of England [19], though the authors concluded that they suffer from ‘inadequate staffing’, 96% of the program reported multi-professional teams of greater than five healthcare workers. Dieticians were available in almost 90%, pharmacists in 75%, psychologists in slightly over 30%, in addition to nurses and physiotherapists which were almost always part of the team. In the aspect of the program contents, 86% used some sort of psychological evaluation tools and 79% had stress management program, which seems to be far better than Korean status. However, vocational counseling was only scarcely provided (4%).

National survey in Portugal [20] showed that dieticians were in the CR team in 92% and psychiatrist/psychologist participated in 76% in 25 centers which were directed by cardiologists, though it was not clear whether they are dedicated to CR or part-time. Among components of CR programs, nutritional counseling was available in 100%, smoking cessation counseling in 88%, psychosocial assessment in 68%, vocational counseling 48%, sexual counseling 48%.

A recent survey of 15 centers in India [21] reported that physiotherapists were available mostly (dedicated 66.6%, part-time 26.6%) and nurses 20% dedicated, 33.3% part-time. Proportion of other health professionals in dedicated/part-time position were: dietician 33.3/66.6%, psychologist 6.6/60%, exercise specialist 33.3/6.6%. In the program contents, nutritional counseling was available for 100%, depression screening 80%, psychological counseling 86.6%, smoking cessation sessions 73.3%, vocational counseling 66.6%, stress management 100%. The study described that the earliest program in India began in 1997, which is quite similar to Korea. Though there is apparently very big unmet need of CR programs in India, the quality of established programs in terms of comprehensiveness seems to be superior to Korea which has comparable duration of history of CR program.

Considering the study findings showing inadequacy of the program contents, educational contents should be improved and/or newly introduced to implement more comprehensive CR program. However, patient education in Korean CR program is restricted by National Health Insurance. While reimbursement for exercise sessions and cardiopulmonary function test can be extended when there is a new indication, patient education is reimbursed for only once regardless of the change in the patients’ clinical status [22]. Because the effect of behavioral intervention heavily depends on the method/intensity/duration of the intervention [10], it should be multifaceted [7] and needs multiple educational sessions [23]. Awareness of this perspective should be enhanced, not only in medical and paramedical personnel who are directly involved in patient care, but also in administrators and policy makers.

Inferring from the findings in the Table 4, hospitals *without* CR program seem to have their own educational program though not in the name of CR education. Proportion of providing general education for CV risk management and medication adherence was slightly lower compared to the hospitals with CR and education for exercise was absent in many centers. Introducing exercise-based CR program and integration with preexisting educational program would be a good starting point for these hospitals.

During the period of year 2008–2020, the CR programs in Korea were established as a part of the Regional Cardiovascular Centers (RCC) project. Before RCC project, CR programs were established were mainly in the capital area, so the comparison between the capital area and other regions crudely reflected the difference between the RCC and non-RCC programs. The characteristics of the CR program in the RCC were addressed in previously published article reporting regional difference [14]. This study has several limitations. (1) This is a cross-sectional survey performed in 2020, thus it may not represent the whole status of Korea which is constantly changing. (2) Due to the COVID 19 pandemic, direct visits to the CR centers could not be done. However, because response rate of PCI-performing hospitals was reasonably good (80%), this study may provide a reliable representation of 2020 status of the most CR programs in Korea. The survey was done during 2020 during COVID 19 pandemic, which may influence the organization of the participating hospitals by various factors including shortage of required personnel. While it is not highly likely that there were profound changes in less than one-year period, this is another limitation because we do not have data before pandemic for comparison. (3) Due to the brevity of the questions, details of the program contents could not be grasped. For example, a question regarding the smoking cessation program was absent, which were available in comparable studies in other countries. In Korea, because smoking cessation support program provided by the National Health Insurance has dominated [24], it seems to be rare in Korean context that formal smoking cessation program is a component of CR program though brief education encouraging smoking cessation may be included in an educational session covering CV risk factor management.

## Conclusions

The comprehensiveness of CR program contents is inadequate, and the staffing of relevant professionals for multidisciplinary team is suboptimal in Korea. In addition to the well-known issue of underutilization of CR, effort to improve the quality of the program is warranted by enhancing its comprehensiveness. More awareness of current status by both clinicians and health policy makers is needed and insurance reimbursement for educational program should be improved.

## Data Availability

The datasets generated and/or analyzed during the current study are not publicly available as public data sharing was not approved by IRB but are available from the corresponding author on reasonable request.
